# Mental Health and Suicide Risk Among High School Students and Protective Factors — Youth Risk Behavior Survey, United States, 2023

**DOI:** 10.15585/mmwr.su7304a9

**Published:** 2024-10-10

**Authors:** Jorge V. Verlenden, Ari Fodeman, Natalie Wilkins, Sherry Everett Jones, Shamia Moore, Kelly Cornett, Valerie Sims, Ryan Saelee, Nancy D. Brener

**Affiliations:** ^1^Division of Adolescent and School Health, National Center for Chronic Disease Prevention and Health Promotion, CDC; ^2^Division of Injury Prevention, National Center for Injury Prevention and Control, CDC

## Abstract

Adolescent mental health and suicide risk remain substantial public health concerns. High pre-COVID rates of poor mental health and suicide-related behaviors have continued to rise, highlighting the need to identify factors that might foster positive mental health outcomes and reduce suicide-related behaviors at population levels. Using CDC’s 2023 Youth Risk Behavior Survey, CDC analyzed the prevalence of mental health and suicide risk indicators and their associations with individual-, family-, and school- or community-level protective factors. Prevalence estimates were calculated for each of the mental health and suicide risk indicators by demographic characteristic. Prevalence ratios adjusted for sex, sexual identity, grade, and race and ethnicity were calculated to examine the association between protective factors and mental health and suicide risk indicators. Overall, 39.7% of students experienced persistent feelings of sadness and hopelessness, 28.5% experienced poor mental health, 20.4% seriously considered attempting suicide, and 9.5% had attempted suicide. Mental health and suicide risk indicators differed by sex, sexual identity, grade, and race and ethnicity. All protective factors were associated with lower prevalence of one or more risk indicators. Findings from this report can serve as a foundation for the advancement of research on protective factors and for the development and implementation of programs, practices, and policies that protect and promote mental health and emotional well-being among youth.

## Introduction

Poor mental and behavioral health among adolescents remains a substantial public health concern. High pre-COVID rates of poor mental health and suicide-related behaviors have continued to rise, particularly among certain subgroups of youth such as female and lesbian, gay, bisexual, and questioning (LGBQ+) students (*1*–*3*). In 2021, suicide was the third leading cause of death among U.S. high school youth aged 14–18 years with 1,952 suicide-related deaths resulting in a rate of 9.0 per 100,000 youths (*2*). However, suicide attempts and suicidal thoughts among youth exceed deaths by suicide. The 2021 Youth Risk Behavior Survey (YRBS) found that approximately one third (30.0%) of female high school students and 14.3% of male high school students had seriously considered attempting suicide during the 12 months before the survey (*1*). YRBS data from 2021 also revealed that over 42% of high school students experienced persistent feelings of sadness or hopelessness during the past year, and 29% of high school students reported their mental health in the past 30 days was not good most of the time or always (*1*). In addition, data collected during July 2021–December 2022 on the Teen National Health Interview Survey of adolescents aged 12–17 years estimated that 21% of adolescents reported experiencing symptoms of anxiety in the past 2 weeks and 17% reported experiencing symptoms of depression (*3*).

Healthy People 2030 highlights the need for prevention and health promotion strategies to reduce suicide-related behaviors and improve mental health outcomes at the population level (*4*). YRBS monitors priority health behaviors and experiences and includes questions related to mental health and suicide and exposures to positive experiences and behaviors that might serve as protective factors. Protective factors are broadly defined as behaviors and characteristics of the adolescent’s environment that are associated with decreased likelihood for experiencing negative outcomes or found to mitigate the negative effects of risk factors such as exposure to adversity (*5*). For example, physical activity, positive coping skills, and supportive social networks have been identified as protective factors that can reduce stress and enhance health (*4*,*5*).

This report summarizes 2023 YRBS data regarding mental health and suicide risk and examines associations with individual-, family-, and school- or community-level health-promoting behaviors and experiences (i.e., protective factors). Understanding the association between protective factors and mental health and suicide-related indicators is important for identifying pathways to resiliency and guiding prevention efforts (*4*,*5*). Findings can be used to guide the design of interventions that promote and protect the mental health and well-being of youth and to inform decision-making among public health and educational leaders.

## Methods

### Data Source

This report includes data from the 2023 YRBS (N = 20,103), a cross-sectional, school-based survey conducted biennially since 1991. Each survey year, CDC collects data from a nationally representative sample of public and private school students in grades 9–12 in the 50 U.S. states and the District of Columbia. Additional information about YRBS sampling, data collection, response rates, and processing is available in the overview report of this supplement (*6*). The prevalence estimates for all variables for the surveillance population overall and stratified by demographic characteristics are available at https://nccd.cdc.gov/youthonline/App/Default.aspx. The full YRBS questionnaire, data sets, and documentation are available at https://www.cdc.gov/yrbs/index.html. This activity was reviewed by CDC and was conducted consistent with applicable Federal law and CDC policy.*

### Measures

This analysis examined two indicators of mental health and two indicators of suicide risk. Mental health indicators included 1) persistent feelings of sadness or hopelessness (past 12 months) and 2) poor mental health (always or most of the time, past 30 days). Suicide risk indicators included 1) seriously considered attempting suicide (past 12 months) and 2) attempted suicide (past 12 months). Six protective factors also were examined, including 1) physically active for ≥60 minutes/day ≥5 days, 2) getting ≥8 hours of sleep; 3) household adult tried to meet their basic needs (always), 4) parental monitoring (high), 5) school connectedness (high), and 6) played on ≥1 sports teams (Table 1). All variables were dichotomized and coded with the absence or lower exposure as the reference category.

**TABLE 1:** Question and analytic coding for health behaviors and experiences, by variable assessed—Youth Risk Behavior Survey, United States, 2023

Variable	Question	Response option	Analytic Coding
Mental health and suicide risk indicators			
Persistent feelings of sadness or hopelessness	During the past 12 months, did you ever feel so sad or hopeless almost every day for two weeks or more in a row that you stopped doing some usual activities?	Yes or no	Yes versus no
Poor mental health	During the past 30 days, how often was your mental health not good?	Never, rarely, sometimes, most of the time, or always	Yes (always, most of the time) versus no (sometimes, rarely, never)
Seriously considered attempting suicide	During the past 12 months, did you ever seriously consider attempting suicide?	Yes, no	Yes versus no
Attempted suicide	During the past 12 months, how many times did you actually attempt suicide?	0 times, 1 time, 2 or 3 times, 4 or 5 times, or ≥6 times	Yes (≥1 times) versus no (0 times)
Protective factors			
Individual-level			
Physically active for ≥60 minutes/day ≥5 days	During the past 7 days, on how many days were you physically active for a total of at least 60 minutes per day?	0 days, 1 day, 2 days, 3 days, 4 days, 5 days, 6 days, or 7 days	Yes (≥5 days) versus no (≤4 days)
Getting ≥8 hours of sleep	On an average school night, how many hours of sleep do you get?	4 or less hours, 5 hours, 6 hours, 7 hours, 8 hours, 9 hours, or 10 or more hours	Yes (≥8 hours) versus no (≤7 hours)
Family/household-level			
Adult in your household tried hard to meet basic needs	During your life, how often has there been an adult in your household who tried hard to make sure your basic needs were met, such as looking after your safety and making sure you had clean clothes and enough to eat?	Always, most of the time, sometimes, rarely, or never	Always (always) versus not always (most of the time, sometimes, rarely, never)
Parental monitoring	How often do your parents or other adults in your family know where you are going or with whom you will be?	Never, rarely, sometimes, most of the time, or always	High (always, most of the time) versus low (sometimes, rarely, never)
School or community-level			
School connectedness	Do you agree or disagree that you feel close to people at your school?	Strongly agree, agree, not sure, strongly disagree, or disagree	High (strongly agree, agree) versus low (not sure, strongly disagree, disagree)
Played on ≥1 sports team	During the past 12 months, on how many sports teams did you play? (Count any teams run by your school or community groups.)	0 teams, 1 team, 2 teams, or ≥3 teams	Yes (≥1 team) versus no (<1 team)

Demographic variables included the following: sex (female and male), sexual identity (heterosexual, gay or lesbian, bisexual, questioning [I am not sure about my sexual identity/questioning], and students who describe their sexual identity in some other way [I describe my identity some other way]), grade in school (9, 10, 11, and 12), and race and ethnicity (American Indian or Alaska Native [AI/AN], Asian, Black or African American [Black], Native Hawaiian or other Pacific Islander [NH/OPI], White, Hispanic or Latino [Hispanic], and Multiracial). (Persons of Hispanic origin might be of any race but are categorized as Hispanic; all racial groups are non-Hispanic.)

### Analysis

Descriptive analyses were conducted to determine the prevalence estimates and corresponding 95% CIs for each of the mental health and suicide risk indicators and each of the mental health and suicide risk indicators by each protective factor. Pairwise *t*-tests compared the prevalence of mental health and suicide indicators by demographic characteristic and by each protective factor. All prevalence estimates and measures of association used Taylor Series Linearization. Tests were considered statistically significant at the p<0.05 level.

Adjusted prevalence ratios (aPRs) were calculated using logistic regression with predicted marginals, which controlled for sex, sexual identity, grade, and race and ethnicity, to examine the association between protective factors and mental health and suicide risk indicators. The aPRs were considered statistically significant if the 95% CI did not cross the null value of 1.0. All analyses were conducted using SAS-callable SUDAAN (version 11.0.4; RTI International) to account for the complex sampling design and weighting.

## Results

### Mental Health and Suicide Risk Indicators

Overall, 39.7% of students experienced persistent feelings of sadness and hopelessness, 28.5% experienced poor mental health, 20.4% seriously considered attempting suicide, and 9.5% attempted suicide (Table 2). The prevalence among female students was higher than among male students for persistent feelings of sadness or hopelessness (52.6% versus 27.7%), poor mental health (38.8% versus 18.8%), seriously considered attempting suicide (27.1% versus 14.1%), and attempted suicide (12.6% versus 6.4%). Similarly, the prevalence among LGBQ+ students was higher than among heterosexual students for persistent feelings of sadness or hopelessness (65.7% versus 31.4%), poor mental health (53.5% versus 21.5%), seriously considered attempting suicide (41.0% versus 13.0%), and attempted suicide (19.7% versus 6.0%).

**TABLE 2:** Prevalence of mental health and suicide risk indicators among high school students, by demographic characteristics — Youth Risk Behavior Survey, United States, 2023*

**Characteristic**	**Persistent feelings of sadness or hopelessness^†^** % (95% CI)^§,¶^	**Poor mental health^†^** % (95% CI)^§,¶^	**Seriously considered attempting suicide^†^** % (95% CI)^§,¶^	**Attempted suicide^†^** % (95% CI)^§,¶^
**Sex**				
Female**	52.6 (50.1–55.0)	38.8 (36.2–41.4)	27.1 (24.7–29.6)	12.6 (11.2–14.2)
Male	27.7 (25.9–29.6)	18.8 (17.3–20.5)	14.1 (12.4–15.9)	6.4 (5.3–7.6)
**Sexual Identity**				
Heterosexual	31.4 (29.8–33.0)	21.5 (20.1–22.9)	13.0 (12.1–14.1)	6.0 (5.2–6.8)
LGBQ+^††^	65.7 (63.0–68.3)	53.5 (50.8–56.1)	41.0 (38.2–43.9)	19.7 (17.8–21.8)
**Grade**				
9^§§^	40.3 (37.3–43.4)	27.1 (24.3–30.1)	21.3 (18.7–24.3)	10.4 (9.0–11.9)
10	39.7 (37.1–42.3)	29.4 (26.8–32.2)	19.7 (17.9–21.6)	9.7 (8.2–11.4)
11	39.7 (36.4–43.1)	29.7 (26.6–32.9)	20.3 (17.3–23.5)	9.4 (7.7–11.3)
12	38.8 (35.7–42.0)	27.9 (25.7–30.2)	19.5 (17.0–22.3)	8.0 (6.3–10.2)
**Race and ethnicity** ^¶¶^				
American Indian or Alaska Native***	44.8 (29.0–61.8)	42.3 (23.9–63.2)	24.5 (14.7–38.1)	11.5 (6.8–18.6)
Asian^†††,§§§^	32.1 (28.3–36.2)	23.0 (18.2–28.7)	14.4 (11.9–17.3)	8.0 (5.3–11.7)
Black or African American^¶¶¶^	39.6 (37.1–42.2)	26.5 (23.1–30.2)	19.6 (16.9–22.7)	10.3 (8.5–12.5)
Native Hawaiian or other Pacific Islander	25.8 (14.3–42.1)	14.9 (4.5–39.6)	16.1 (4.3–45.0)	15.3 (4.0–43.8)
White	38.9 (36.4–41.4)	31.4 (29.0–33.9)	22.1 (19.8–24.5)	8.3 (7.0–9.9)
Hispanic or Latino****^,††††^	42.4 (39.4–45.4)	26.1 (23.2–29.2)	18.2 (16.2–20.3)	10.8 (9.1–12.6)
Multiracial^§§§§^	41.4 (35.2–47.8)	28.9 (24.1–34.2)	21.6 (17.3–26.7)	11.4 (8.4–15.5)
**Total**	**39.7 (37.7–41.7)**	**28.5 (26.7–30.4)**	**20.4 (18.7–22.3)**	**9.5 (8.4–10.7)**

* N = 20,103 respondents. The total number (N) of students answering each question varied. Data may be missing because 1) the question did not appear in that student’s questionnaire, 2) the student did not answer the question, or 3) the response was set to missing because of an out-of-range response or logical inconsistency. Percentages in each category are calculated on the known data.

^†^ Refer to Table 1 for variable definitions.

^§^ Percentages in each category are calculated on the known data.

^¶^ All prevalence estimates and measures of association used Taylor Series Linearization. Tests were considered statistically significant at the p<0.05 level.

** Female students had significantly different prevalences than male students on all mental health and suicide risk indicators (i.e., persistent feelings of sadness or hopelessness, poor mental health, seriously considering attempting suicide, suicide attempts).

^††^ LGBQ+ students had significantly different prevalences than heterosexual students on all mental health and suicide risk indicators (i.e., persistent feelings of sadness or hopelessness, poor mental health, seriously considering attempting suicide, suicide attempts).

^§§^ 9th Graders had significantly different prevalence of suicide attempts than 12th graders.

^¶¶^ Persons of Hispanic origin might be of any race but are categorized as Hispanic; all racial groups are non-Hispanic.

*** American Indian or Alaska Native had significantly different prevalence of poor mental health than Native Hawaiian or Other Pacific Islander (NH/OPI) students.

^†††^ Asian students had a significantly different prevalence of persistent feelings of sadness or hopelessness and seriously considered attempting suicide than Black, Hispanic, Multiracial, and White students.

^§§§^ Asian students had a significantly different prevalence of poor mental health than White students.

^¶¶¶^ Black students had a significantly different prevalence of poor mental health and attempted suicide than White students.

**** Hispanic students had a significantly different prevalence of persistent feelings of sadness or hopelessness than NH/OPI and White students.

^††††^ Hispanic students had a significantly different prevalence of poor mental health, seriously considered attempting suicide, and attempted suicide than White students.

^§§§§^ Multiracial students had a significantly different prevalence of persistent feelings of sadness or hopelessness than NH/OPI students.

Mental health and suicide risk indicators also differed by grade and by race and ethnicity (Table 2). The prevalence of attempted suicide was higher among students in 9^th^ grade compared with students in 12^th^ grade (10.4% versus 8.0%). Persistent feelings of sadness or hopelessness, poor mental health, seriously considered attempting suicide, and attempted suicide varied by race and ethnicity, but no consistent patterns emerged, with various significantly different group comparisons on different risk indicators. For example, compared with White students, Hispanic students had greater prevalence of persistent feelings of sadness or hopelessness (42.4% versus 38.9%) and attempted suicide (10.8% versus 8.3%). Conversely, White students had greater prevalence of poor mental health (31.4% versus 26.1%) and of seriously considering attempting suicide (22.1% versus 18.2%) compared with Hispanic students. Similarly, compared with White students, Black students had lower prevalence of poor mental health (26.5% versus 31.4%), but they had higher prevalence of attempted suicide (10.3% versus 8.3%). Asian students had lower prevalence of seriously considering attempting suicide compared with Black, Hispanic, multiracial, and White students (14.4% versus 19.6%, 18.2%, 21.6%, and 22.1% respectively).

### Mental Health and Suicide Risk Indicators by Protective Factors

All protective factors were significantly associated with lower prevalence of one or more mental health and suicide risk indicators (Figure). Being physically active for ≥60 minutes/day on ≥5 days was associated with lower prevalence of having experienced persistent feelings of sadness or hopelessness (aPR = 0.92) (Supplementary Table, https://stacks.cdc.gov/view/cdc/160632). Getting ≥8 hours of sleep was associated with lower prevalence of all mental health and suicide risk indicators (aPR range = 0.53–0.67) as was having a household adult who always tried to meet their basic needs (aPR range = 0.41–0.80). High parental monitoring was associated with lower prevalence of all mental health and suicide risk indicators (aPR range = 0.51–0.74) except poor mental health. High levels of school connectedness were associated with lower prevalence of all mental health and suicide risk indicators (aPR range = 0.63–0.70). Playing on ≥1 sports team was associated with lower prevalence of all mental health and suicide risk indicators (aPR range = 0.84–0.90) except attempted suicide.

**FIGURE:**
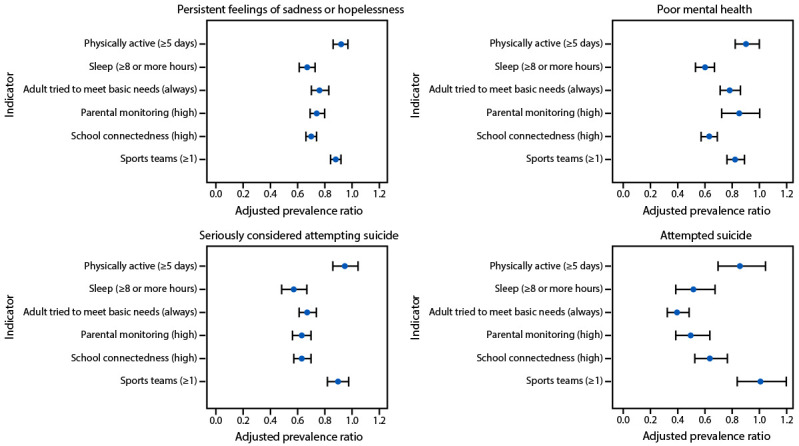
Adjusted prevalence ratio* of mental health and suicide risk indicators, by individual-level, household-level, and school or community-level protective factors**^†^** — Youth Risk Behavior Survey, United States, 2023^§^ * Adjusted prevalence ratios were calculated using logistic regression with predicted marginals, controlled for sex, sexual identity, grade, and race and ethnicity. Adjusted prevalence ratios were considered statistically significant if the 95% CIs did not cross the null value of 1.0. Bars indicate 95% CI. ^†^ Refer to Table 1 for variable definitions. ^§^ N = 20,103 respondents. The total number (N) of students answering each question varied. Data may be missing because 1) the question did not appear in that student’s questionnaire, 2) the student did not answer the question, or 3) the response was set to missing because of an out-of-range response or logical inconsistency. Percentages in each category are calculated on the known data.

## Discussion

Overall, results from the 2023 YRBS illustrate the high prevalence of mental health and suicide-related risk indicators among U.S. adolescents. Nearly one in three students experienced poor mental health most of the time or always during the 30 days before the survey, over one in three students felt persistent sadness or hopelessness for 2 weeks or more during the 12 months before the survey, one in five students seriously considered attempting suicide during the 12 months before the survey, and nearly one in 10 students attempted suicide during the 12 months before the survey. The prevalence of mental health and suicide-related risk indicators was high across all demographic groups; however, prevalence was highest among female students and LGBQ+ students. Compared with male students, female students were about twice as likely to have experienced each mental health and suicide risk indicator, and compared with their heterosexual peers, LGBQ+ students were two to three times as likely to have experienced the risk indictors measured.

Such disparities among girls and LGBQ+ youth have been highlighted previously (*1*–*3*). The past six cycles of YRBS data (2011–2021) have shown female students with higher prevalence of persistent feelings of sadness or hopelessness and higher prevalence of suicide risk indicators than male students (*1*). Likewise, examination of emergency department (ED) usage data collected in the 2018 to 2021 National Hospital Ambulatory Medical Care Survey (NHAMCS) and data from the National Syndromic Surveillance Program have described sex differences in ED visits related to mental health where adolescent girls were treated for mental health disorders such as depression, anxiety, and trauma and stressor-related disorders at higher rates than boys (*7*,*8*). For girls, research has shown differences in the mental health impact of academic stress, cultural expectations, social media use, and violence (*9*). In addition, as described in previous YRBS surveillance reports (*1*), findings from analysis of 2023 YRBS data indicated that LGBQ+ students were more likely to experience mental health and suicide risk than their heterosexual peers. Stress associated with rejection, marginalization, and discrimination and with trauma related to personal victimization contribute to depressive symptoms, suicide risk, and other disparities in behavioral health outcomes for LGBQ+ youth (*10*).

Differences by race and ethnicity were identified for each mental health and suicide risk indicator. For example, Hispanic/Latino students were more likely to report persistent feelings of sadness or hopelessness during the past year and were also more likely to have attempted suicide than White students. However, White students reported a higher prevalence of suicidal ideation during the past year compared with Asian and Hispanic/Latino students. Demographic differences need further investigation because research has documented disparities in mental health outcomes and suicide risks for racial and ethnic minority adolescents. For example, 2018–2021 NHAMCS data indicated that mental health related ED visits were higher among Black youths than Hispanic and White youths (*7*). In addition, the prevalence of seriously considered attempting suicide increased significantly during 2019–2021 among Black, Hispanic, and White female students and among Hispanic male students (*1*). The nuanced patterns found in 2023 YRBS data, combined with patterns of increased risk for negative mental health and suicide-related outcomes among racial and ethnic minority students shown in previous studies (*1*–*3*,*7*,*8*), underscore the importance of culturally tailored prevention interventions, including suicide awareness and mental health literacy efforts that reduce stigma and support help seeking (https://www.cdc.gov/minority-health/features/minority-mental-health.html).

Findings from this report suggest ways that individual behaviors, household experiences, and community/school influences might attenuate mental health and suicide risk and might facilitate trajectories that lead to positive health behaviors and outcomes. In this analysis, students who reported being physically active ≥60 minutes on ≥5 days, getting adequate sleep ≥8 hours, having a household adult who always tried to meet their basic needs, high levels of parental monitoring, high levels of school connectedness, and playing on a sports team had lower prevalence of at least one mental health and suicide risk indicator, which is consistent with previous research.

Certain studies have documented the positive effects of physical activity on self-esteem, relationships, academic achievement, and physical and mental health (https://health.gov/healthypeople/tools-action/browse-evidence-based-resources/physical-activity-guidelines-americans-2nd-edition). In addition, physical activity and attending physical education classes during an average week have been associated with higher levels of feeling close to persons at school, which has protective effects related to emotional well-being along with potential to prevent and mitigate health risks (*5*). However, previous research indicates that not all students have equal access or opportunities for engaging in physical activity (*11*). For example, LGBQ+ students have reported lower levels of physical activity because of feeling uncomfortable and unsafe in physical activity-related settings (e.g., physical education classes and locker rooms) (*12*).

The relation between sleep and mental health also is well documented. For adolescents, adequate sleep (getting ≥8 or more hours) is critical for cognitive development and emotional well-being (*5*), whereas insufficient sleep (getting <8 hours) can compromise functioning across several domains, including emotional regulation, mood, and stress reactivity (*13*). The findings in this report indicate that getting the recommended ≥8 hours of sleep was associated with a lower prevalence of all mental health and suicide risk indicators, underscoring the benefits of adolescents getting adequate sleep. However, many adolescents get less than the recommended hours of sleep (*13*). Further research examining factors that influence adolescent sleep patterns and strategies for improving sleep duration and quality (e.g., school start times, quantity of homework, students’ technology and social media use, afterschool activities, and employment responsibilities) are needed to identify ways to bolster this foundational protective factor.

Consistent with previous studies on parental monitoring and adolescent mental health and suicide outcomes, high parental monitoring was associated with lower risk for persistent feelings of sadness and hopelessness, suicidal thoughts, and suicide attempts (*14*). Previous research indicates that the extent to which parents are aware of adolescents’ whereabouts is influenced by both parental behaviors (e.g., soliciting information) and adolescent perspectives (e.g., relationship satisfaction with parent) (*15*). As such, higher levels of parental monitoring might indicate more positive communication and connectedness between adolescents and their parents, which in turn has been linked to reduced risk for mental health and suicide outcomes (*15*).

Having a household adult that always tried to meet basic needs was associated with a lower prevalence of all mental health and suicide risk indicators. Caregiver nurturance and stable caregiving have been found to moderate academic, social, and psychological resilience in youth (*5*). This is especially evident among youth exposed to concentrated disadvantage, such as exposure to community violence and being in foster care (*5*,*16*).

The findings in this report also highlight the potential benefits to mental health of playing on a school or community sport team. Other research indicates that participation in team sports might promote mental health because of opportunities to build social relationships that foster a sense of connection and belonging (*17*). However, cost, time, and lack of inclusive spaces are barriers to team sport involvement that have been highlighted previously and elevate the need for more affordable and inclusive local opportunities for youth to participate (*11*,*12*,*18*).

Finally, school connectedness was associated with lower risk for all mental health and suicide indicators. This adds to previous evidence on the important role that safe and supportive school environments play in supporting students’ mental health and well-being during adolescence and into adulthood (*5*,*19*). However, research has also shown that female, Black, Hispanic, and LGBQ+ youth and youth who have experienced racism feel less connected to school than their peers, highlighting the importance of creating opportunities for connectedness and belonging for students at higher risk for feeling marginalized at school (*19*).

## Limitations

General limitations for the YRBS are available in the overview report of this supplement (*6*). The findings in this report are subject to at least four additional limitations. First, causality and direction of associations between protective factors and student behaviors and experiences cannot be inferred by these cross-sectional data. Referring to variables as “protective factors” denotes a conceptual naming convention, backed by extant literature; however, because temporality cannot be parsed out from these surveillance data, these factors might not always occur before the outcome. Second, individual-level behaviors, household experiences, school or community influences, and mental health and suicide risk indicators have differing recall periods and different time reference points, which might contribute to recall bias and affect validity of constructs and comparability of items. Third, the interrelated qualities of protective factors cannot be disentangled with these data; therefore, their independent contribution to an association with a mental health or suicide risk indicator cannot be assessed. Finally, socioeconomic status of a student’s household and community have been shown to modify the relation between protective factors and mental health indicators; however, these variables could not be accounted for because they are not measured in the survey.

## Future Directions

The high prevalence of poor mental health and suicide risk indicators among female students and LGBQ+ students and differences by race and ethnicity underscore the urgency for comprehensive research to explore factors that contribute to these disparities. Such work is critical to informing the design of public health interventions and programming to reduce suicide risk and improve mental health outcomes for youth. In addition, because of the importance of protective factors in improving health, even in the face of risk behaviors and negative experiences, it is important to consider monitoring additional protective factors in future survey research. Research also is needed to examine the dimensions of protective constructs used in this analysis through more nuanced, multi-item measures and scales, along with relations between protective factors. Finally, further examination of programs, initiatives, or interventions is necessary to ensure they promote protective experiences and address barriers to access across subpopulations, which might be essential for meeting the needs of adolescents at the highest risk of negative outcomes.

## Conclusion

During 2023, significant differences in mental health outcomes and suicidal thoughts and behaviors among youth were observed across sex, sexual identity, and race and ethnicity. The findings in this report also found that protective factors at the individual-, family-, school-, and community-levels were associated with decreased mental health and suicide risk among high school students. Findings from this report can serve as a foundation for the advancement of research on associations between protective factors and mental health among youth. Results highlight the need for the development and implementation of inclusive, culturally, and linguistically appropriate programs, practices, and policies that protect and promote mental health and emotional well-being among youth. Moreover, as part of a larger comprehensive suicide prevention approach, strategies that both reinforce youth health-promoting behaviors and experiences at multiple levels and consider the role of cultural differences across demographic groups might be more effective in bolstering youth mental health and reducing suicidal thoughts and behaviors. CDC’s Promoting School Mental Health and Wellbeing: An Action Guide for School and District Leaders (https://www.cdc.gov/healthyyouth/mental-health-action-guide/index.html), the What Works in Schools program (https://www.cdc.gov/healthyyouth/whatworks/index.htm), and the Suicide Prevention Resource for Action (https://www.cdc.gov/suicide/resources/prevention.html) provide strategies for school, district, and community leaders based on the best available evidence for promoting mental health and emotional well-being and preventing suicide.

## Footnotes

* 45 C.F.R. part 46.114; 21 C.F.R. part 56.114.
